# Knowledge and Attitude Toward Evidence-Based Medicine and Associated Factors Among Medical Interns in Amhara Regional State Teaching Hospitals, Northwest Ethiopia: Cross-sectional Study

**DOI:** 10.2196/28739

**Published:** 2021-06-24

**Authors:** Delelegn Emwodew, Tesfahun Melese, Adamu Takele, Nebiyu Mesfin, Binyam Tariku

**Affiliations:** 1 School of Public Health Dilla University Dilla Ethiopia; 2 Institute of Public Health University of Gondar Gondar Ethiopia; 3 School of Medicine University of Gondar Gondar Ethiopia

**Keywords:** knowledge, attitude, evidence-based medicine, teaching hospitals

## Abstract

**Background:**

Evidence-based medicine (EBM) is widely accepted in medicine. It is necessary to improve the knowledge and attitudes of medical students in the use of evidence. In Ethiopia, little is known about medical students’ knowledge and attitudes toward EBM.

**Objective:**

This study aimed to assess the knowledge and attitudes toward EBM and its associated factors among medical interns in teaching hospitals.

**Methods:**

A cross-sectional survey was conducted using a random sample of medical interns in teaching hospitals in Ethiopia. Multivariable logistic regression analyses were used to identify the factors associated with the knowledge and attitudes toward EBM. Adjusted odds ratio (AOR) with 95% confidence interval and *P*≤.05 was used to quantify strength of association between variables.

**Results:**

Out of a sample of 423 medical interns, 403 completed the questionnaire (95.3% response rate). Overall, 68.0% (274/403 of respondents had a favorable attitude toward EBM and 57.1% (230/403) had good knowledge of EBM. The majority (355/403, 88.1%) of participants had internet access. Only 19.6% (79/403) of respondents had received EBM-related training. Respondents’ knowledge of EBM was associated with previous EBM training (AOR 2.947, 95% CI 1.648-5.268, *P*<.001), understanding of sensitivity (AOR 2.836, 95% CI 1.824-4.408, *P*=.003), and internet access (AOR 2.914, 95% CI 1.494-5.685, *P*=.002). The use of an electronic database as a source of information (AOR 1.808, 95% CI 1.143-2.861, *P*=.01) and understanding of absolute risk reduction (AOR 2.750, 95% CI 1.105-6.841, *P*=.03) were predictors of positive attitudes.

**Conclusions:**

This study demonstrates a lack of formal EBM training and awareness of basic concepts of EBM among medical interns. Medical intern attitudes toward EBM are relatively good. To enhance EBM knowledge and skills, formal teaching of EBM should be integrated into medical education.

## Introduction

Evidence-based medicine (EBM) is the systematic identification, evaluation, and use of up-to-date research evidence as the basis for clinical decisions [1]. The practice of EBM means integrating clinical expertise and the best available evidence with the ideas, concerns, and expectations of individual patients [2,3]. It includes 5 steps: formulating clinical questions, finding and retrieving evidence, critically appraising evidence, applying evidence, and evaluating performance [4]. EBM has the potential to improve the continuity and uniformity of care through the development of common approaches and guidelines [5,6]. It can help clinicians make better use of limited resources by enabling them to evaluate the clinical- and cost-effectiveness of treatments and services [6].

Several studies have examined the familiarity, awareness, and attitudes toward EBM among medical students [7,8]. A study conducted in Iran shows that most medical students do not have enough knowledge of basic concepts and familiarity with the term EBM [9]. Similarly, poor EBM knowledge among medical students was found in Switzerland [10]. The attitude toward EBM was generally positive among medical and health science students in Hungary [11]. In contrast, medical students’ knowledge and attitude toward EBM were low in Saudi Arabia [12].

Most medical schools teach EBM as a major component of their medical curriculum [13,14]. “In Africa, EBM is emphasized in countries such as South Africa, Ethiopia, Kenya, Nigeria, Egypt, Botswana, Burundi, and Malawi” [15,16]. Several universities in different African countries offer EBM courses, but most are located in South Africa [15]. Most Ethiopian physicians have unmet training needs concerning EBM and seek support for an improved education system to provide quality evidence-based health care [17,18].

For the training of physicians, it is essential to perform needs assessments and evaluate the level of knowledge and their attitudes. “In Ethiopia, EBM teaching has not yet become part of the undergraduate medical education, which has led to gaps in knowledge, skills, and attitudes toward EBM. Particularly, little is known about medical students’ knowledge and attitudes toward EBM [19]. Therefore, the current state of EBM must be understood to plan long-term educational programs” [20]. Our study aimed to assess knowledge and attitudes toward EBM and associated factors among medical interns in teaching hospitals in northwestern Ethiopia.

## Methods

### Study Area and Period

The study was conducted at the University of Gondar and Tibebe Ghion teaching hospitals in the Amhara region of the northwestern province of Ethiopia between March and April 2020. The Amhara region is located in the northwestern and northern parts of Ethiopia. It has 10 administrative zones, 181 woredas (districts), and 78 urban centers. According to the 2019 Amhara Regional Health Office, there are two teaching hospitals in the area, the University of Gondar and Tibebe Ghion. These teaching hospitals are training centers for undergraduate medical students and others who are responsible for solving public health problems across the country.

### Study Design and Population

A cross-sectional study design was employed. Participants of this study were all medical interns in teaching hospitals of the northwestern province of Ethiopia. Medical interns who were on sick leave and those with a week off during the study period were excluded.

### Sample Size and Sampling Procedure

The sample size was determined using the single population proportion formula n = (Zα/2) 2*P* (1–p) / d2; where, n = sample size, Zα/2 (1.96): significance level at α=.05, p: proportion for knowledge and attitude = 50%, and d: margin of error (.05). After adding a 10% nonresponse rate, the final sample size was 423.

By taking a list of medical interns from each hospital, we determined the proportionate sample to be taken to estimate the number of study participants per hospital using the formula = (n)×(nf) / N where n = the number of medical interns at each hospital, nf = total sample size, and N = total number of medical interns at the two hospitals. After that, based on their population, a simple random sampling method was used.

### Measurements and Data Collection Methods

Data were collected using a 6-section self-administered questionnaire: sociodemographic information, EBM knowledge, attitudes toward EBM, information source preferences, awareness of EBM resources, and understanding of statistical terms. The questionnaire was taken from previous studies because these previous works had already been validated [21,22]. Data were collected by 6 health informatics (BSc) students. Respondents’ attitudes were rated on 11 questions on 5-point Likert scales. All individual responses to attitudinal questions were calculated to obtain total scores; after that, the mean score was calculated to be classified as favorable (if respondents scored average or above) or unfavorable (if participants received less than average score). Also, knowledge level was measured by calculating the average value of 14 items and was rated as good (if respondents scored the mean value or above of correctly replied questions) or poor (if participants scored below the mean score of correctly replied questions).

### Data Quality Assurance and Management

One-day training was provided for the data collectors and supervisors on how the data should be obtained and recorded. Ongoing follow-up and supervision were performed by supervisors and the principal investigator throughout the data collection process. The data were checked daily for completeness and consistency.

A pretest of the questionnaire was conducted prior to the actual data collection in 5% of the sample in Tikur Anbessa Hospital, which was not included in the study. Using data obtained from the pretest, the questionnaire was tested for reliability (internal consistency) using the Cronbach alpha test. The reliability of the knowledge questions had a Cronbach alpha value of .84 and the attitude had a Cronbach alpha value of .76. These figures indicate that the questionnaire is very reliable.

### Data Processing and Analysis

The data collected was coded and entered in the freely available public health software Epi Info version 7.1 and analyzed using SPSS (version 20.1, IBM Corp). Descriptive analysis was used to compute the mean, standard deviation, frequency, and percentage of each variable. The Spearman rank correlation coefficient test was used to test the bivariate correlation between outcome and predictor variables. The choice of variables to be included in the final model was made by examining the correlations between the independent variables to remove the variables with the strong association. Finally, multivariable logistic regression analysis was used to identify factors associated with the knowledge and attitude of medical interns. Factors with significant associations were identified based on adjusted odds ratio (AOR) with a 95% confidence interval and *P*≤.05.

### Ethical Statement

This study was approved by the University of Gondar Institute of Public Health Research Ethics Committee (no. IPH837) and followed the guidelines of the Helsinki Declaration. All participants were given written informed consent before enrollment in the study. Medical interns were informed of their absolute right to skip any questions or not participate in the study. Confidentiality was maintained throughout the study, and respondents were assured that the results would be used for research purposes only.

## Results

### Sociodemographic Characteristics

From a sample of 423 medical interns, 403 completed a questionnaire (response rate 95.3%). Of the 403 participants in the study, 291 (72.2%) were male. The majority (296/403, 73.4%) of participants were from the University of Gondar teaching hospital, and the remainder were from Tibebe Ghion teaching hospital. Most (324/403, 80.4%) of the respondents had never received EBM-related training. Most (276/403, 68.5%) of the participants had a computer and 88.1% (355/403) had access to the internet (Table 1).

**Table 1:** Sociodemographic characteristics of medical interns in northwest Ethiopia in 2020 (n=403).

Variables			University of Gondar (n=296), n (%)		Tibebe Ghion (n=107), n (%)
**Sex**					
	Male	221 (74.4)		70 (65.4)	
	Female	75 (25.3)		37 (34.6)	
**Previous EBM^a^** **training**					
	Yes	65 (22.0)		14 (13.1)	
	No	231 (78.0)		93 (86.9)	
**Have a computer**					
	Yes	210 (70.9)		66 (61.7)	
	No	86 (29.1)		41 (38.3)	
**Have internet access**					
	Yes	259 (87.5)		96 (89.7)	
	No	37 (12.5)		11 (10.3)	

^a^EBM: evidence-based medicine.

### Knowledge About EBM

Of the participants, 57.1% (230/403) had a good knowledge of EBM with a mean score of 6.6 (SD 3.62). Just over half (226/403, 56.1%) of the participants responded correctly that EBM is a combination of good research evidence and clinical expertise. Similarly, 55.1% (222/403) were aware of the need for critical assessment skills to assess the quality of research papers, and 52.6% (212/403) agreed that the EBM practice required proper identification and clinical questioning. However, only 26.8% (108/403) responded correctly that a literal search using medical subject heading (MeSH) terms would reveal fewer articles than the actual search using a simple keyword (Table 2).

**Table 2:** Knowledge of EBM among medical interns in northwest Ethiopia, 2020 (n=403).

Knowledge assessment items	Correct, n (%)	Incorrect, n (%)
EBM^a^ is the integration of best research evidence with clinical expertise and patient values and preferences.	226 (56.1)	177 (43.9)
A literature search using MeSH^b^ terms would yield fewer articles than a basic search using general terms.	108 (26.8)	295 (73.2)
A literature search using the Boolean operator “OR” would reduce the number of citations.	108 (26.8)	295 (73.2)
Research using clinical trials is generally more reliable than research using the observational method.	210 (52.1)	193 (47.9)
Clinical trials and observational methods are equally valid in establishing treatment effectiveness.	183 (45.4)	220 (54.6)
Evidence and patients are equally important to make clinical decisions.	184 (45.7)	219 (54.3)
Evidence alone is not enough to make a good clinical decision.	222 (55.1)	181 (44.9)
Within EBM, expert opinion is not considered as a form of evidence.	147 (36.5)	256 (63.5)
The practice of EBM requires the appropriate identification and formulation of clinical questions.	212 (52.6)	191 (47.4)
An etiological question is best answered through the use of a cohort study.	189 (46.9)	214 (53.1)
In therapy questions, a randomized controlled trial provides the best information to make a good clinical decision.	196 (48.6)	207 (51.4)
Understanding of patient preferences is essential for identifying the best available treatment.	217 (53.8)	186 (46.2)
EBM requires the use of critical appraisal skills to ensure the quality of all the research papers retrieved.	222 (55.1)	181 (44.9)
Critically appraised evidence should be appropriately applied to the patient using clinical experience.	231 (57.3)	172 (42.7)

^a^EBM: evidence-based medicine.

^b^MeSH: medical subject heading.

### Attitude Toward EBM

Among the total participants, 68.0% (274/403) had a favorable attitude toward EBM with an attitude mean score of 9.7 (SD 1.65). The majority (380/403, 94.3%) of participants believe that the EBM practice is a useful tool for clinical decision making and 91.3% (368/403) agreed that the EBM practice improves patient care (Table 3).

**Table 3:** Attitude toward EBM among medical interns in northwest Ethiopia, 2020 (n=403).

Attitude assessment items	Agree, n (%)	Disagree, n (%)
Using results from research is important for the development of my professional practice.	382 (94.8)	21 (5.2)
The practice of EBM^a^ is a helpful tool for decision making in my clinical practice.	380 (94.3)	23 (5.7)
The practice of EBM helps me to care for people in the same way and with the same efficiency.	313 (77.7)	90 (22.3)
The practice of EBM improves the quality of my work.	350 (86.8)	53 (13.2)
The practice of EBM can reduce health care cost.	302 (74.9)	101 (25.1)
The application of EBM is necessary for my work.	365 (90.6)	38 (9.4)
The practice of EBM improves patient care.	368 (91.3)	35 (8.7)
I believe EBM improves the quality and results of my clinical interventions.	358 (88.8)	45 (11.2)
I consider research findings useful in my daily practice.	358 (88.8)	45 (11.2)
I am interested in learning or improving the skills necessary to incorporate EBM into my work.	371 (92.1)	32 (7.9)
I need to increase the use of evidence in my daily work.	383 (95.0)	20 (5.0)

^a^EBM: evidence-based medicine.

### Preference of Information Sources

Most (366/403, 90.8%) participants had read the medical textbook in search of information. Also, 81.4% (328/403) of participants consulted colleagues and 75.0% (306/403) consulted senior doctors when seeking information. However, only 40.4% (163/403) had read articles found by searching an electronic database to guide their clinical decision (Table 4).

**Table 4:** Preference of information sources among medical interns in northwest Ethiopia, 2020 (n=403).

Information sources	Yes, n (%)	No, n (%)
Read medical textbook.	366 (90.8)	37 (9.2)
Read printed research articles.	125 (31.0)	278 (69.0)
Refer to clinical practice guidelines.	255 (63.3)	148 (36.7)
Read articles found by searching of electronic databases.	163 (40.4)	240 (59.6)
Refer to medical apps.	222 (55.1)	181 (44.9)
Consult colleagues.	328 (81.4)	75 (18.6)
Consult senior doctors.	306 (75.9)	97 (24.1)

### Awareness of EBM Resources

Only a minority of respondents were aware of EBM resources (Table 5). Some participants were aware of PubMed (56/403, 13.9%), Clinical Evidence (from BMJ Publishing Group; 35/403, 8.7%), and Cochrane Database of Systematic Reviews (26/403, 7.2%). Only a few knew about Bandolier (20/403, 5.0%) and the Database of Abstracts of Reviews of Effects (DARE; 14/403, 3.5%).

**Table 5:** Awareness of EBM resources among medical interns in northwest Ethiopia, 2020 (n=403).

Evidence-based medicine resources	Aware, n (%)	Unaware, n (%)
Centre for Evidence-Based Medicine	21 (5.2)	382 (94.8)
American College of Physicians Journal Club	20 (5.0)	383 (95.0)
Cochrane Database of Systematic Reviews	29 (7.2)	374 (92.8)
Database of Abstracts of Reviews of Effects	14 (3.5)	389 (96.5)
Bandolier	20 (5.0)	383 (95.0)
PubMed	56 (13.9)	347 (86.1)
Clinical Evidence (from BMJ Publishing Group)	35 (8.7)	368 (91.3)
Evidence-Based Medicine (from BMJ Publishing Group)	19 (4.7)	384 (95.3)

### Understanding Statistical Terms

Only some of the respondents had an understanding of statistical terms used in EBM such as relative risk (150/403, 37.2%), *P* value (145/403, 36.0%), sensitivity (143/403, 35.5%), and others. Despite this, the medical interns had a good understanding of relative risk reduction (47/403, 11.7%) and absolute risk reduction (40/403, 9.9%; Table 6).

**Table 6:** Understanding of statistical terms among medical interns in northwest Ethiopia, 2020 (n=403).

Statistical terms	Understand, n (%)	Don’t understand, n (%)
Absolute risk reduction	40 (9.9)	363 (90.1)
Relative risk reduction	47 (11.7)	356 (88.3)
Number needed to treat	58 (14.4)	345 (85.6)
Confidence interval	132 (32.8)	271 (67.2)
*P* value	145 (36.0)	258 (64.0)
Sensitivity	143 (35.5)	260 (64.5)
Specificity	138 (34.2)	265 (65.8)
Likelihood ratio	91 (22.6)	312 (77.4)
Relative risk	150 (37.2)	253 (62.8)
Odds ratio	140 (34.7)	263 (65.3)

### Factors Associated With EBM Knowledge

In bivariate analysis, variables such as having a computer (*P*=.001), EBM training (*P*=.000), internet access (*P*=.000), awareness of Bandolier (*P*=.006), awareness of PubMed (*P*=.001), awareness of DARE (*P*=.004), understanding of relative risk reduction (RRR) (*P*=.001), understanding of sensitivity (*P*=.000), and understanding of odds ratio (*P*=.000) indicate significant associations at *P*<.01 significance level.

In the multivariable analysis, EBM training, internet access, PubMed awareness, understanding of sensitivity, and RRR showed significant association with medical interns’ knowledge of EBM. The chances of having good EBM knowledge among medical interns who took EBM training were 2.9 times (AOR 2.947, 95% CI 1.648-5.268) higher than those who did not take EBM training. Medical interns with internet access were 2.9 times (AOR 2.914, 95% CI 1.494-5.685) more likely to have better EBM knowledge compared to those without internet access.

Respondents who knew PubMed were 2.9 times (95% CI 1.4-6.0) more likely to have a better knowledge of EBM than those who did not. Participants who understood sensitivity were 2.8 times (AOR 2.836, 95% CI 1.824-4.408) more likely to have a good EBM knowledge compared to those who did not understand the sensitivity. Medical interns who understood the RRR were 2.7 times (AOR 2.760, 95% CI 1.85-6.431) more likely to have a good EBM knowledge compared to those who did not (Table 7).

**Table 7:** Factors associated with EBM knowledge among medical interns in northwest Ethiopia, 2020 (n=403).

Variable		Knowledge level			COR^a^ (95% CI)		AOR^b^ (95% CI)		*P* value
		Poor, n (%)	Good, n (%)						
**EBM^c^ training**		—^d^	—	3.1 (1.7-5.5)		2.9 (1.6-5.2)		<.001	
	No	155 (89.6)	169 (73.5)	—		—		—	
	Yes	18 (10.4)	61 (26.5)	—		—		—	
**Internet access**		—	—	3.4 (1.8-6.4)		2.9 (1.5-5.7)		.002	
	No	33 (19.1)	15 (6.5)	—		—		—	
	Yes	140 (89.9)	215 (93.5)	—		—		—	
**PubMed**		—	—	3.6 (1.5-7.2)		2.9 (1.4-6.0)		.003	
	Unaware	162 (93.6)	185 (80.4)	—		—		—	
	Aware	11 (6.4)	45 (19.6)	—		—		—	
**Sensitivity**		—	—	2.9 (1.8-4.5)		2.8 (1.8-4.4)		.000	
	Don’t understand	134 (77.5)	126 (54.8)	—		—		—	
	Understand	39 (22.5)	104 (45.2)	—		—		—	
**Relative risk reduction**		—	—	4.2 (1.9-9.3)		2.7 (1.8-6.4)		.019	
	Don’t understand	165 (95.4)	191 (83.0)	—		—		—	
	Understand	8 (4.6)	39 (17.0)	—		—		—	

^a^COR: crude odds ratio.

^b^AOR: adjusted odds ratio.

^c^EBM: evidence-based medicine.

^d^Not applicable.

### Factors Associated With Attitude Toward EBM

In the bivariate analysis, the use of an electronic database (*P*=.002) showed significant correlations at the *P*<.01 level. EBM knowledge (*P*=.02) and absolute risk reduction (ARR) (*P*=.01) understanding indicate significant association at *P*<.05 levels. In the multivariable analysis, EBM knowledge, understanding of ARR, and the use of electronic databases were factors significantly associated with attitudes toward EBM.

The probability of having a positive EBM attitude among medical interns who understood ARR was 2.7 times (AOR 2.750, 95% CI 1.105-6.841) higher than those not understood. Respondents who used an electronic database to make clinical decisions were 1.8 times (AOR 1.808, 95% CI 1.143-2.861) more likely to have a positive attitude toward EBM compared to those who did not use electronic databases. Participants with good EBM knowledge were 1.6 times (AOR 1.610, 95% CI 1.004-2.493) more likely to have a positive attitude toward EBM compared to those with less EBM knowledge (Table 8).

**Table 8:** Factors associated with attitude toward EBM among medical interns in northwest Ethiopia, 2020 (n=403).

Variable		Attitude level			COR^a^ (95% CI)		AOR^b^ (95% CI)		*P* value
		Unfavorable, n (%)	Favorable, n (%)						
**EBM^c^ knowledge**		—^d^	—	1.6 (1.1-2.5)		1.6 (1.0-2.5)		.03	
	Poor	66 (51.2)	107 (39.1)	—		—		—	
	Good	63 (48.8)	167 (60.9)	—		—		—	
**Use electronic database**		—	—	2 (1.3-3.1)		1.8 (1.1-2.9)		.01	
	No	91 (70.5)	149 (54.4)	—		—		—	
	Yes	38 (29.5)	125 (45.6)	—		—		—	
**Absolute risk reduction**		—	—	2.9 (1.2-7.1)		2.7 (1.1-6.8)		.03	
	Don’t understand	123 (95.3)	240 (87.6)	—		—		—	
	Understand	6 (4.7)	34 (12.4)	—		—		—	

^a^COR: crude odds ratio.

^b^AOR: adjusted odds ratio.

^c^EBM: evidence-based medicine.

^d^Not applicable.

## Discussion

### Principal Findings

The results of this study revealed that medical interns have limited knowledge of the basics of EBM but have relatively positive attitudes. We have found medical interns more widely used and more reliant on printed textbooks and consulting with senior doctors when seeking information. Also, they had little awareness of EBM resources and lacked sufficient understanding of statistical terms. The output from the multivariable analysis identified EBM training, internet access, awareness of PubMed, understanding of sensitivity, and RRR as significant predictors of medical interns’ knowledge of EBM.

This study showed that only 13.9% of participants knew about PubMed. Similarly, some studies have shown a low level of awareness of EBM resources among medical students [9,12]. Also, only 8.7% of medical interns were aware of Clinical Evidence (from the BMJ Publishing Group). In contrast, a study on knowledge, attitudes, and barriers to EBM in residents reported that 31.6% of participants knew about the Clinical Evidence website [23]. This difference may be because the concept of EBM is still a new term among the medical interns included in this study. This has shown that the teaching hospitals included in this study have done little to raise awareness among medical interns about EBM resources.

Correspondingly, the findings from this study show that 90.1% of participants did not understand ARR and 85.6% do not understand the number needed to treat. This could be because the majority (80.4%) of participants included in this study did not have any EBM-related training. This has shown that little has been done to increase knowledge and skills about EBM among medical students. Therefore, medical students should be trained about statistical terms used in EBM.

In this study, 57.1% of participants had a good knowledge of EBM. Nearly half (55.1%) of respondents correctly answered that critical appraisal skills are necessary to ensure the quality of research papers, and 52.6% correctly answered that EBM practice requires proper identification and formation of clinical questions. This is consistent with a study of EBM in medical students in Switzerland [24]. In contrast, a survey of knowledge, attitudes, and behaviors of medical students in Ireland showed that almost all (97%) participants were aware of the need for critical appraisal skills to ensure the quality of all research papers, and the majority (94%) were aware that the EBM practice required proper identification and clinical questioning [25]. These differences may be due to the lack of formal EBM training in teaching hospitals included in this study. Experimental evidence from Mexico suggested that the formal student training in EBM improved the knowledge and skills of medical students with EBM [26].

The results of this study revealed that the majority (68.0%) of participants had a positive attitude toward EBM. These findings were consistent with evidence from other studies [27]. This could be the first step in motivation and was a good sign to promote EBM teaching in the medical student curriculum. Similarly, a study conducted on knowledge and attitudes of EBM among Jordanian physicians showed that 63.5% of participants had a positive attitude of EBM [28]. In contrast, a study conducted among Saudi Arabian medical students reported unwelcome attitudes toward EBM [12]. Of our participants, 91.3% agreed that practicing EBM improves patient care. Similarly, a study conducted on the knowledge and attitude of EBM in Iran has shown that 92.6% of physicians believe that practicing EBM improves patient management [29]. Also, 94.3% of participants included in our study believed that the EBM practice was a useful tool for clinical decision making. This was higher than a study conducted on the knowledge and attitude of evidence-based practice in which 80% of participants believed that EBM helps with treatment decisions.

Several factors affected EBM knowledge: internet access, EBM training, PubMed awareness, and familiarity with sensitivity. This finding was consistent with previous studies in Ethiopia, which identified lack of training as the most significant factor associated with physicians’ and nurses’ knowledge of EBM [30]. A study conducted in Saudi Arabia also found a significant increase in knowledge between academic levels, seminar attendees, and nonattendees [31]. The study also found that EBM knowledge, awareness of ARR, and the use of the electronic database as a source of information were factors that positively affected medical intern attitudes toward EBM. An observational study from Yemen found a significant association of age with the positive attitude of physicians toward EBM, while this study found no significant association [32]. The diﬀerence between studies could be attributed to diﬀerent samples as this study included medical interns who are in a similar age group.

The concept of EBM was still unfamiliar among medical interns included in this study due to a lack of formal training. This will require a national policy for the EBM program in medical universities and needs to be addressed at all levels of medical education in Ethiopia. Numerous studies have shown that incorporating EBM into the medical curriculum enhances medical students’ skill in forming clinical questions, searching for evidence, and evaluating the evidence [33-36]. Also, medical students’ knowledge and attitudes improved after EBM training [26,37].

### Strength and Limitations

The strong point of the study was that the survey tool was adapted from a previously used and standardized EBM measurement tool. This is the first study to investigate medical interns’ knowledge and attitude of EBM in teaching hospitals of northwest Ethiopia. There are some limitations to this study. First, it is subject to questionnaire study and response bias. We were able to reduce response bias by getting very good response rates. Second, the study was conducted only in teaching hospitals, which may affect the generalizability of the findings to other settings. Finally, there were few women in the sample, which may be due to the small number of female students at medical universities in Ethiopia.

### Conclusions

Attitudes toward EBM were often favorable among medical interns in northwestern Ethiopia. However, the interns lacked appropriate EBM training, awareness of EBM resources, and understanding of methodological terms. This information will help in providing appropriate practical training on EBM and enable medical interns to apply EBM when making treatment decisions to provide the best medical care for patients. Teaching hospitals should teach EBM to undergraduate medical students to improve the quality of health care and ensure that students have the knowledge and skills needed to use EBM in actual clinical practice.

Therefore, it is recommended that EBM be included in a variety of teaching activities such as small group teaching, task assignments, morning seminars, and ward rounds with ongoing assessment by academic instructors. In addition, training students to thoroughly search EBM resources such as electronic databases of systematic reviews on a daily or weekly basis is essential for teaching. Additional studies are needed to assess the level of knowledge and attitudes of medical students about EBM at Ethiopian medical universities.

## Appendix

Multimedia Appendix 1 Survey questionnaire.

## Abbreviations

AOR: adjusted odds ratio
ARR: absolute risk reduction
EBM: evidence-based medicine
DARE: Database of Abstracts of Reviews of Effects
MeSH: medical subject heading
RRR: relative risk reduction

## References

[1] Straus S, McAlister F (2000). Evidence-based medicine: a commentary on common criticisms. CMAJ.

[2] Maggio LA, Kung JY (2014). How are medical students trained to locate biomedical information to practice evidence-based medicine? A review of the 2007-2012 literature. J Med Libr Assoc.

[3] Sackett D, Rosenberg WM, Gray JA, Haynes RB, Richardson WS (1996). Evidence based medicine: what it is and what it isn't. BMJ.

[4] Dawes M, Summerskill W, Glasziou P, Cartabellotta A, Martin J, Hopayian K, Porzsolt F, Burls A, Osborne J, Second International Conference of Evidence-Based Health Care Teachers and Developers (2005). Sicily statement on evidence-based practice. BMC Med Educ.

[5] Al Omari M, Khader Y, Jadallah K, Dauod A, Al-Shdifat A (2009). Awareness, attitude and practice of evidence-based medicine among primary health care doctors in Jordan. J Eval Clin Pract.

[6] Lewis S, Orland B (2004). The importance and impact of evidence-based medicine. J Manag Care Pharm.

[7] Alahdab F, Firwana B, Hasan R, Sonbol MB, Fares M, Alnahhas I, Sabouni A, Ferwana M (2012). Undergraduate medical students' perceptions, attitudes, and competencies in evidence-based medicine (EBM), and their understanding of EBM reality in Syria. BMC Res Notes.

[8] Ilic D, Forbes K (2010). Undergraduate medical student perceptions and use of Evidence Based Medicine: a qualitative study. BMC Med Educ.

[9] Ghahremanfard F, Nassaji M, Mirmohammadkhani M, Tanha A, Mosavi M, Ghaemi A, Shams P (2014). Knowledge and attitude toward evidence-based medicine among medical students in Semnan, Iran. J Evid Based Med.

[10] Kaderli R, Burghardt L, Hansali C, Businger A (2012). Students' view of evidence-based medicine: a survey in Switzerland. Arch Clin Exp Surg.

[11] Csertő M, Berényi K, Decsi T, Lohner S (2019). Self-reported attitudes, knowledge and skills of using evidence-based medicine in daily health care practice: a national survey among students of medicine and health sciences in Hungary. PLoS One.

[12] Aldugieman T, Alanezi R, Alshammari W, Al-Shamary Y, Alqahtani M, Alreshidi F (2018). Knowledge, attitude and perception toward evidence-based medicine among medical students in Saudi Arabia: analytic cross-sectional study. J Family Med Prim Care.

[13] Lai NM, Teng CL (2011). Self-perceived competence correlates poorly with objectively measured competence in evidence based medicine among medical students. BMC Med Educ.

[14] Tilson JK, Kaplan SL, Harris JL, Hutchinson A, Ilic D, Niederman R, Potomkova J, Zwolsman SE (2011). Sicily statement on classification and development of evidence-based practice learning assessment tools. BMC Med Educ.

[15] Forland F, Rohwer AC, Klatser P, Boer K, Mayanja-Kizza H (2013). Strengthening evidence-based healthcare in Africa. Evid Based Med.

[16] Law T, Lavis J, Hamandi A, Cheung A, El-Jardali F (2012). Climate for evidence-informed health systems: a profile of systematic review production in 41 low- and middle-income countries, 1996-2008. J Health Serv Res Policy.

[17] Abdulwadud O, Tadesse F, Yilma G, Midekssa M, Ibraghimova I (2018). Knowledge and experience with Cochrane and evidence based medicine among health professionals in Debreberhan Referral Hospital in Ethiopia: a cross-sectional survey. Pan Afr Med J.

[18] Worku T, Yeshitila M, Feto T, Leta S, Mesfin F, Mezmur H (2019). Evidence-based medicine among physicians working in selected public hospitals in eastern Ethiopia: a cross-sectional study. BMC Med Inform Decis Mak.

[19] Balajić K, Barac-Latas V, Drenjancević I, Ostojić M, Fabijanić D, Puljak L (2012). Influence of a vertical subject on research in biomedicine and activities of The Cochrane Collaboration branch on medical students' knowledge and attitudes toward evidence-based medicine. Croat Med J.

[20] Okoromah CAN, Adenuga AO, Lesi FEA (2006). Evidence-based medicine curriculum: impact on medical students. Med Educ.

[21] Fernández-Domínguez J, de Pedro-Gómez J, Morales-Asencio JM, Bennasar-Veny M, Sastre-Fullana P, Sesé-Abad A (2017). Health Sciences-Evidence Based Practice questionnaire (HS-EBP) for measuring transprofessional evidence-based practice: creation, development and psychometric validation. PLoS One.

[22] Larsen C, Terkelsen A, Carlsen A, Kristensen H (2019). Methods for teaching evidence-based practice: a scoping review. BMC Med Educ.

[23] Al Wahaibi A, Adawi S, Shehhi W, Rizvi SG, Al-Kemyani N, Al-Amrani K, Al-Khabori M (2014). Knowledge and attitudes of Oman medical specialty board residents towards evidence-based medicine. Oman Med J.

[24] Rohwer A, Young T, van Schalkwyk S (2013). Effective or just practical? An evaluation of an online postgraduate module on evidence-based medicine (EBM). BMC Med Educ.

[25] Stronge M, Cahill M (2012). Self-reported knowledge, attitudes and behaviour towards evidence-based practice of occupational therapy students in Ireland. Occup Ther Int.

[26] Sánchez-Mendiola M, Kieffer-Escobar L, Marín-Beltrán S, Downing SM, Schwartz A (2012). Teaching of evidence-based medicine to medical students in Mexico: a randomized controlled trial. BMC Med Educ.

[27] Abeysena C, Jayawardana P, Wickremasinghe R, Wickramasinghe U (2010). Evidence-based medicine knowledge, attitudes, and practices among doctors in Sri Lanka. J Evid Based Med.

[28] Barghouti F, Halaseh L, Said T, Mousa A, Dabdoub A (2009). Evidence-based medicine among Jordanian family physicians: awareness, attitude, and knowledge. Can Fam Physician.

[29] Rashidbeygi M, Sayehmiri K (2013). Knowledge and attitudes of physicians towards evidence based medicine in Ilam, Iran. Iran Red Crescent Med J.

[30] Abdulwadud O, Azazh A, Mekasha A, Heye TB, Nigatu B, Debebe F, Emiru HG (2019). Cochrane, evidence-based medicine and associated factors: a cross-sectional study of the experiences and knowledge of Ethiopian specialists in training. Afr J Emerg Med.

[31] Bindawas SM (2013). Evidence-based health care continuing education seminars improve academic staff knowledge and attitudes in Saudi Arabia. Pak J Med Sci.

[32] Bin Briek A, Webair HH, Al-Tuhaify MM (2014). Assessment of physicians' attitude, awareness and knowledge of evidence-based medicine: an observation from Yemen. J Fam Med.

[33] Ahmadi S, Baradaran HR, Ahmadi E (2015). Effectiveness of teaching evidence-based medicine to undergraduate medical students: a BEME systematic review. Med Teach.

[34] Dorsch JL, Aiyer MK, Meyer LE (2004). Impact of an evidence-based medicine curriculum on medical students' attitudes and skills. J Med Libr Assoc.

[35] Mitku A, Dessie Z, Muluneh E, Workie D (2016). Prevalence and associated factors of TB/HIV co-infection among HIV infected patients in Amhara region, Ethiopia. Afr Health Sci.

[36] Sastre EA, Denny JC, McCoy JA, McCoy AB, Spickard A (2011). Teaching evidence-based medicine: impact on students' literature use and inpatient clinical documentation. Med Teach.

[37] Khader Y, Batayha W, Al-Omari M (2011). The effect of evidence-based medicine (EBM) training seminars on the knowledge and attitudes of medical students towards EBM. J Eval Clin Pract.

