# Supplementary material for: Knowledge and Attitude Toward Evidence-Based Medicine and Associated Factors Among Medical Interns in Amhara Regional State Teaching Hospitals, Northwest Ethiopia: Cross-sectional Study
Source: JMIR Med Educ. 2021 Jun 24;7(2):e28739. doi: 10.2196/28739 (PMC8277356; doi:10.2196/28739)
Supplement: Multimedia Appendix 1 [file mededu_v7i2e28739_app1.docx]

**Multimedia Appendix 1: Survey questionnaire**

**Part 1: Socio-demographic information**

This section of the questionnaire asks about your demographic profile. For each item, please circle your response.

| No | Questions | Response option |
| --- | --- | --- |
| 1 | Sex | 1. Male 2. Female |
| 2 | Age | –––––years |
| 3 | Where is your current place of studying? | 1. University of Gondar teaching hospital 2. Tibebe-Ghion teaching hospital |
| 4 | Do you have an own computer? | 1. Yes 2. No |
| 5 | Have you ever received any training related to EBM? | 1. Yes 2. No |
| 6 | Do you have internet access? | 1. Yes 2. No |

**Part 2: EBM knowledge**

This part of the questionnaire is designed to gather information about your EBM knowledge. For each item, please locate the X in the box corresponding to your answers.

| No | Questions | Yes | No | Don’t know |
| --- | --- | --- | --- | --- |
| 1 | Evidence-based medicine (EBM) is the integration of best research evidence with clinical expertise and patients values and preferences in making clinical decisions about their care |  |  |  |
| 2 | Literature search on Medline using MeSH (medical subject headings) terms would yield fewer articles than a basic search using general terms |  |  |  |
| 3 | Literature search using Boolean operator ‘OR’ would reduce the number of citations that the search would produce |  |  |  |
| 4 | Research using clinical trials is generally more reliable than research using the observational method |  |  |  |
| 5 | Clinical trials and observational methods are equally valid in establishing treatment effectiveness |  |  |  |
| 6 | Evidence and patients are equally important to make clinical decisions |  |  |  |
| 7 | Evidence alone is not enough to make a good clinical decision |  |  |  |
| 8 | Within EBM, expert opinion is not considered as a form of evidence |  |  |  |
| 9 | The practice of EBM requires the appropriate identification and formulation of clinical questions |  |  |  |
| 10 | An etiological question is best answered through the use of cohort study |  |  |  |
| 11 | In therapy questions, randomized control trial (RCT) provide the best information to make a good clinical decision |  |  |  |
| 12 | Understanding of patient’s preferences are essential for identifying the best available treatment for that particular patient |  |  |  |
| 13 | EBM requires the use of critical appraisal skills to ensure the quality of all the research papers retrieved |  |  |  |
| 14 | Critically appraised evidence should be appropriately applied to the patient using clinical judgment and experience |  |  |  |

**Part 3: Attitudes toward EBM**

The purpose of this part of the questionnaire is to find out your opinion about EBM. For each item, please mark the X on the box corresponding to your responses.

| No | Questions | Strongly agree | Agree | Neutral | Disagree | Strongly disagree |
| --- | --- | --- | --- | --- | --- | --- |
| 1 | Using results from research is important for the development of my professional practice |  |  |  |  |  |
| 2 | The practice of EBM is a helpful tool for decision-making in my clinical practice |  |  |  |  |  |
| 3 | The practice of EBM helps me to care for people in the same way and with the same efficiency |  |  |  |  |  |
| 4 | The practice of EBM improves the quality of my work |  |  |  |  |  |
| 5 | The practice of EBM can reduce healthcare cost |  |  |  |  |  |
| 6 | The application of EBM is necessary in my work |  |  |  |  |  |
| 7 | The practice of EBM improves patient care |  |  |  |  |  |
| 8 | I consider EBM improves the quality and results of my clinical interventions |  |  |  |  |  |
| 9 | I consider research findings are useful in my daily practice |  |  |  |  |  |
| 10 | I am interested in learning or improving the skills necessary to incorporate EBM into my work |  |  |  |  |  |
| 11 | I need to increase the use of evidence in my daily work |  |  |  |  |  |

**Part 4:** **Preferences of information sources**

This section of the questionnaire asks your preferences for information resources to make a clinical decision. For each item, please mark the X on the box corresponding to your responses.

| No | Questions | Yes | No |
| --- | --- | --- | --- |
| 1 | Read medical textbook |  |  |
| 2 | Read printed research articles |  |  |
| 3 | Refer clinical practice guidelines |  |  |
| 4 | Read articles from searching of electronic databases |  |  |
| 5 | Refer medical apps |  |  |
| 6 | Consult colleagues |  |  |
| 7 | Consult senior doctors |  |  |

**Part 5:** **Awareness of EBM resources**

This part of the questionnaire asks about your awareness of some of the resources available and used in the EBM. For each item, please mark the X on the box corresponding to your responses.

| No | Questions | Aware | Unaware |
| --- | --- | --- | --- |
| 1 | Centre of Evidence-based medicine (CEBM) |  |  |
| 2 | American College of Physician (ACP) Journal Club |  |  |
| 3 | Cochrane database of Systematic Review |  |  |
| 4 | Database of abstracts of reviews of effectiveness (DARE) |  |  |
| 5 | Bandolier (Published in Oxford) |  |  |
| 6 | PUBMED/Medline journal |  |  |
| 7 | Clinical evidence (from the BMJ Publishing group) |  |  |
| 8 | Evidence based medicine (from the BMJ Publishing Group) |  |  |

**Part 6:** **Understanding of statistical terms** **used** **in** **EBM**

This section of the questionnaire asks about your understanding of the terms commonly used in EBM. For each item, please mark the X on the box corresponding to your responses.

| No | Questions | Understand | Don’t understand |
| --- | --- | --- | --- |
| 1 | Absolute risk reduction |  |  |
| 2 | Relative risk reduction |  |  |
| 3 | Number needed to treat |  |  |
| 4 | Confidence interval |  |  |
| 5 | P-value |  |  |
| 6 | Sensitivity |  |  |
| 7 | Specificity |  |  |
| 8 | Likelihood ratio |  |  |
| 9 | Relative risk |  |  |
| 10 | Odds ratio |  |  |
